# Improving Detection of Client Complexity in the Community (Impact): A Study Protocol of a Pragmatic Randomized Controlled Trial

**DOI:** 10.3390/mps4040070

**Published:** 2021-10-06

**Authors:** Jennifer Boak, Irene Blackberry, Tshepo Rasekaba

**Affiliations:** 1 Community Nursing Services, Bendigo Health, Kennington, VIC 3550, Australia; 2 John Richards Centre for Rural Ageing Research, La Trobe University, Wodonga, VIC 3689, Australia; i.blackberry@latrobe.edu.au (I.B.); t.rasekaba@latrobe.edu.au (T.R.)

**Keywords:** home nursing, aged, assessment, health care needs

## Abstract

Background: Community-dwelling older clients are becoming increasingly complex. Detecting this complexity in clinical practice is limited, with greater reliance on community nurses’ clinical judgment and skills. The lack of a consistent approach to complexity impacts the level of care and support for older clients to remain in their homes for longer. Objective: To examine the effectiveness of the Patient Complexity Instrument (PCI) in addition to nurses’ clinical judgment to enhance detection of complexity, and subsequent older clients’ resource allocation compared to usual nursing assessment. Design: A pragmatic randomized controlled trial will be conducted within a community nursing service in regional Victoria, Australia. Clients 65 years and over referred to the service who are eligible for Commonwealth Home Support Programme (CHSP) funding will be randomized into Control group: usual nursing assessment or Intervention group: usual nursing assessment plus the PCI. Nurse participants are Registered Nurses currently employed in the community nursing service. Results: This study will explore whether introducing the PCI in a community nursing service enhances detection of complexity and client care resource allocation compared to nurses’ clinical judgment based on usual nursing assessment. Conclusion: This protocol outlines the study to enhance the detection of complexity by nurses delivering care for community-dwelling older people in the regional Australian context. The findings will inform the use of a standardized tool to detect complexity among community-dwelling older Australians.

## 1. Introduction

### 1.1. Global Ageing Population and the Implications

Countries around the world, including Australia, are faced with an increasing ageing population. Trending indicates that by 2050, “one person in every five will be over 60 years of age or older” [1]. This demographic shift impacts on the number of people needing support to remain at home. As people age, if their choice is to stay at home, many will require more support. Their needs may increase with varying degrees of complexity depending on their ailments, social situation, access to resources and safety of their environment [2]. Therefore, along with the increasing number of people needing support, their care needs are more complex. 

As new models for chronic conditions and post-discharge care options increase, people are not staying in hospital for as long [3,4,5,6]. People are being discharged with a higher level of care required, with a consequence of increasing complexity of the community-dwelling older people [7,8,9,10] and increased reliance on caregivers for support [11]. Huber et al. recognized that the increasing prevalence of multimorbidity is also impacting care in the community [10]. The flow-on effect is increasing workload pressure on the community nursing services to deliver the complex care to maintain the independence of older people in the community and safety at home.

Internationally, strategies are being developed to support growing ageing populations and improve their health care. Australia, United Kingdom and Canada employ similar strategies including improving access to home and community aged care, support for carers, and reducing the need for residential aged care [12,13,14]. These governments recognize the impact of an ageing population, increase in chronic disease prevalence and the need to think differently about how and where healthcare is delivered. The Royal Commission into Aged Care Quality and Safety in Australia reported that caring for older people in acute hospitals and residential aged care is costly on the healthcare system and there is a need to better develop community programs to support people who wish to remain independent at home [15,16].

### 1.2. Ageing Population in Australia and the Implications

In Australia in 2015, the number of people aged 65 years and over accounted for 15% of the population [17]. Around six out of ten people aged 65 years and over experience 33% of the burden of ill health and say they are in less than very good health [17].). This data has contributed to changes implemented in the health care sector in general. One area of change has been the care of older people in the community. The Australian Government recognized the challenges of an ageing population that is rapidly changing and established the CHSP in 2011 [18]. With less affordable residential care places available in regional and rural areas, there is an 84% increase of people requiring home care over the past 10 years [19]. In 2018 the Australian Government launched additional initiatives aimed at keeping older people well at home and engaged in the community, whilst reducing the need for hospital admission and delaying admission to residential aged care [18,19,20,21]. These initiatives built on strategies implemented since 2011 and have been reviewed again in the national health care reform agreement and 2020–2025 addendum [14,22]. In order to support the CHSP and subsequent initiatives, The National Screening and Assessment (NSAF) was implemented in Australia in 2018 and consisted of two types of home-based assessments [23]. The first is a home support assessment and second is an aged care comprehensive assessment. The home support assessment is required for a client to be eligible for CHSP services and the comprehensive assessment is required for home care package eligibility. The NSAF is a useful starting point for client assessment however, it is difficult to develop rapport in one visit and determine all care required. The nursing assessment using the NSAF may need to be conducted over 2–3 visits to develop a more comprehensive picture of how the client is managing.

### 1.3. Factors Contributing to Complexity

The World Health Organization (WHO) identified that “by the age of 60 years, the major burden of disability and death arises from age-related losses in hearing, seeing and moving, and conditions such as dementia, heart disease, stroke, chronic respiratory disorder, diabetes and musculoskeletal conditions such as osteoarthritis and back pain” [24]. The prevalence of multimorbidity (two or more diseases) is high among older people with 74.6% have multiple chronic conditions [25]. Multimorbidity adds another layer to complexity because of the need for various treatments and coordination of multiple care services. As well as multimorbidity, socioeconomic position, social support options, psychological stress, inadequate nutritional intake, reduce physical activity all have an impact on the family’s ability to assist and the variety of services needed to support the older person [26,27]. These factors influence health outcomes and require comprehensive assessment and monitoring [28].

Ageing is an accumulation of molecular and cellular damage over time which leads to gradually decreasing physical and mental capacity, while increasing risk of diseases and eventually, death [29]. WHO reported that “the loss of ability typically associated with ageing is only loosely related to a person’s chronological age” [29]. Yet, some older people continue to enjoy very good health and function independently while others are frail and require significant supports. The presence of one or more of mobility loss, malnutrition, visual impairment and hearing loss, cognitive impairment or depressive symptoms can be identified as contributing to a declining physical and/or mental capacity [24]. These factors contribute to client complexity and the ability to continue to do what they value most.

Before complexity is measured, it needs to be defined. An overview of the literature indicates that complexity is sometimes used interchangeably with frailty, yet they are different [30]. Frailty is described as a physical wellbeing state, linked to fatigue, resistance and aerobic capability, other illnesses, loss of weight and the client’s awareness of their health [31]. However, this description does not take into consideration personal and environmental factors. Gobbens et al. suggest that frailty could also be considered dynamic when these additional factors are considered [32]. Encompassing the physical as well as psychosocial and environmental aspects of a person is what makes complexity dynamic, meaning that the complex client can shift between recovery and relapse into a complex state [30]. Some definitions of frailty are outdated and place greater emphasis on the medical model view of frailty. More recent definitions consider the multidimensional nature of frailty [33]. In contrast, definitions of complexity are fewer, and there is an increasing reference to the need to determine a client’s complexity. Determining the level of complexity involves conducting a comprehensive assessment that also considers the clients’ level of engagement, clinical need, social contact, family/carers, resources, and safety [2].

Nurses working in the community report that clients are becoming more complex; hence more resources and time are required for nurses to deliver the appropriate care. Contributing to this complexity are factors affecting family structures and supports. For instance, children or family members are no longer live within proximity to their parents or older family members to render assistance or they work full-time to support their own immediate families. Older people wish to continue to live in and actively contribute to their community, and families support the idea of their older parents remaining at home for as long as possible. Current practice and systems in the care of older people need to change from the traditional provider-centered model to a value-based model the enables the older person and their family to be more involved and share their needs and priorities [34,35]. All these factors combined impact a client’s ability to manage independently. A change in one or more of these factors, e.g., the loss of a partner or a new diagnosis, can have an impact on the level of care required, hence the dynamism of client complexity.

### 1.4. Assessment of Older People by Nurses

Nurse-led assessments in the community result in decisions made and actions planned with the older client, regarding referrals and additional resources. The client’s General Practitioner (GP) is informed of community nurse-led assessment but is not the decision maker of care coordination. This contrasts with the bulk of assessments for older people where nurses either independently or as part of a multidisciplinary team collect data but are involved in the care decision making. This is an important difference to other geriatric assessments conducted by nurses such as the primary care assessment for people 75 years and older [36] where the GP makes decisions with the client. InterRAI is a subscription based comprehensive suite of assessments that can be completed by any trained health professional [37], this tool has been adapted for use in home care settings in recent years. There are also recent studies with nurses completing the geriatric assessment data, however the geriatrician will make decisions regarding actions [38,39,40]. In Australian community-based programs, multidisciplinary teams are growing but there is limited access to geriatricians. The assessment tool, clinical judgment clinical experience is important in the decision-making process for complex clients.

Given the range of factors that need to be considered when determining the care needs of an older person, different approaches to assessment have evolved [41]. Comprehensive assessment and monitoring require standardized, robust, and multidimensional tools to assist community nurses to detect client complexity. Such tools need to also consider psychosocial factors and biomedical factors to enable streamlining of clinical care and supportive resources required for the complex client. Clinical judgment is also recognized as an important factor in determining the care needs of an older person [41]. Structured assessment tools alone do not encourage a person-centered model, as clinical judgment is required to link all the aspects of a structure assessment together. 

A scoping literature review was conducted to identify a potential standardized tool to trial. The review methodology and results will be published elsewhere. In summary, a search was performed in Medline and CINAHL. Five tools were identified but only one, the Patient Complexity Instrument (PCI) [2], met the criteria. 

### 1.5. Aim and Objectives

The Improving Detection of Patient Complexity in the Community (ImPaCt) is a trial of the PCI within the community nursing service to improve detection of client complexity in regional Victoria. The primary objective is to explore whether adding the PCI to the usual assessment process could enhance the detection of the complexity of clients and support the clinical judgment. The secondary objective is to explore whether using the PCI derived client complexity could facilitate appropriate resource allocation; these resources being the time allocated to providing clinical care to the complex client or referrals completed to support the client. The study will also explore issues around the feasibility and acceptability of the PCI in community nursing.

This study aims to answer the question “Does the PCI in addition to usual assessment, enhance nurses’ detection of complexity and delivery of appropriate allocation of care for community dwelling patients aged 65 and over?”.

The primary null hypothesis is the addition of the PCI has no effect on nurses’ detection of patient complexity compared to usual assessment alone. The secondary null hypothesis is the addition of the PCI does not enhance the allocation of appropriate resources according to the client’s complexity. 

Ethical approval has been provided by the researchers’ employer and the supporting University reference number (blinded). The trial has been registered with the Australian and New Zealand Clinical Trials Registry (blinded).

## 2. Methods

### 2.1. Study Design

The ImPaCt study is a parallel group blocked randomized controlled trial (RCT) with nested feasibility in which the PCI plus usual assessment of client complexity will be compared to usual assessment method alone. Blocked randomization will ensure a balance in the number of participants between the study groups in the trial and the next participant group allocation is less predictable, reducing the risk of selection bias [42,43]. The data will follow the CONSORT guideline including the CONSORT checklist [43].

### 2.2. Setting and Participants

The PCI will be trialed in a regional Victorian community nursing service, Australia, located at Bendigo Health. The community nursing service is funded by the Australian CHSP to provide traditional district nursing care to clients residing within a radius of 30 km from the service center. Clients are eligible for the service if aged 65 years old and over, have physical and cognitive function limitations, require assistance to remain living independently at home and remain engaged in their community [18]. Approximately 60 newly referred clients are seen monthly in the service by a team of 34 nurses who work a two-shift roster per day for seven days. At any one time, there are over 500 clients using the service. Community-dwelling client are referred to the service from My Aged Care, hospitals, doctors, other health practitioners, family members or themselves. Common reasons for referral are personal care, medication management, wound care, dementia support, diabetes management or continence management. Upon receiving the referral, the Intake Nurse triages the referral, collates all available information, and assigns a nurse who will visit the client for initial face-to-face assessment. A predetermined amount of time is allocated to completing the initial assessment and the associated clinical care. However, the assessment nurse may adjust this time once the assessment has been completed, for subsequent visits, based on the nurse’s assessment of care needs and nurses’ clinical judgment of level client complexity. Even though the information collected in the assessment is comprehensive, interpretation of the information is inconsistent, unstandardized and largely subjective. The decision regarding client complexity is reliant on the skill and clinical judgment by nurses.

Newly referred client assessment will form the unit of evaluation in this study. Nurses who perform these assessments will also be included as participants over the period from 1 July 2019–30 September 2020.

### 2.3. Inclusion Criteria

The inclusion criteria are new referrals of clients aged 65 and over and meet the criteria for CHSP funded services as explained above. All Registered Nurses employed as District Nurses in the community nursing service and are allocated a new client assessment during the trial period will be included. Assessments will be those performed during weekdays.

### 2.4. Exclusion Criteria

Exclusions will be client referrals for specific episodic care such as anti-coagulant therapy or eye drops post-eye surgery. Nurse participation is voluntary therefore, assessments completed by a nurse who declines to participate in the study will not be considered.

### 2.5. Intervention

In this proposed study, the intervention will be made up of the PCI plus usual care assessment of complexity. The PCI measures complexity across six domains engagement in care planning, clinical need (physical and psychological), social contact, family and carers, resources, and safety. Items are scored on a Likert scale of 1 to 5 and are added to give a total score from 6 (low complexity) to 30 (high complexity). The PCI was developed in a community nursing service caring for adults in Wales and validated using Rasch analysis to determine the probability of an item indicating complexity. Each item in the PCI was determined to be a good fit to determine complexity.

### 2.6. Sample Size

Initial data will be used to conduct a post hoc sample size calculation. Up to *n* = 34 (all) nurses in the community nursing service will make up the nurse participant sample. These are nurses making up the team who would normally be available to conduct assessments on referred clients. Based on the number of new referrals expected over three-month period, a convenience sample of up to *n* = 180 patient assessments (90 per group) will be targeted and up to *n* = 27 (all) nurses in the service will make up the nurse participant sample. Due to the pilot nature of the study, formal sample size calculation is not critical. Pilot studies by their nature are not aimed at establishing a definitive effect, however a rationale for sample size basis such as described above is deemed sufficient [44,45].

**Figure 1:**
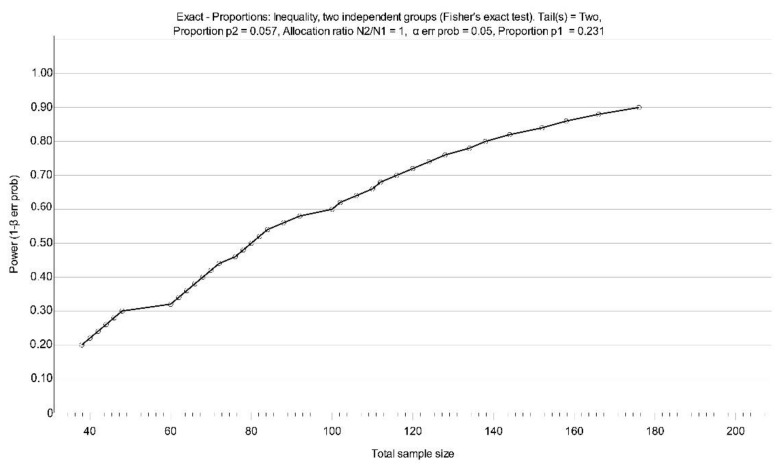
Projected sample size at different study power levels.

Using our early pilot data, post hoc sample size calculation was performed based on the proportions of the level of complexity rating in the ImPaCt study. A minimum total sample *n* = 138 patient assessments, i.e., 69 per group (Figure 1) will be required in definitive trial to detect a significant difference in proportions relating to a high level of complexity rating between the control (23.1%) vs. intervention (5.7%), odds ratio = 4.97, power = 0.8 and *p* < 0.05.

### 2.7. Randomization

The study randomization schedule will be designed by an independent person who will not be implementing the study procedures. Randomization will occur at the level of the type of assessment (PCI plus usual assessment vs. usual assessment alone) using 45 blocks of 4 block sizes.

Allocations will be concealed in sequentially numbered, sealed opaque envelopes prepared by an independent person. An envelope will be sequentially drawn from a closed box and opened to reveal the assessment allocation, each time a new assessment needs to be performed. Once opened the allocation cannot be re-used if for whatever reason it is not used for that assessment.

### 2.8. Study Procedures

Prior to participating in the study, nurses will be briefed about the project and provided with a brief education about using the PCI. This will ensure that each nurse receives the same information. No further instruction will be provided on the PCI which will remove the risk of researcher influence.

Each nurse will be allocated a code that will be used for each assessment they complete. This code will not identify the nurse and will be provided by an independent person, it will be used for analysis purposes only. The code will be allocated to each nurse involved using a sealed envelope system at the commencement of the project and on consent. This will ensure that data related to the learning effect can be analyzed.

The nurses will be provided with an introduction to the PCI and asked to complete the nurse pre RCT survey (File S1 in Supplementary). On completion of data collection, the nurse will be asked to complete the post RCT survey (File S2 in Supplementary).

As described earlier in local context, the Intake Nurse is responsible for allocating new referrals for assessment to nurses through a daily list. They will in addition provide the sealed group allocation envelope to the nurse on return to the office. On return, the nurse will open the envelope to determine the group type. The nurse will then retrieve the appropriate group envelope containing the required study documents. The control group envelope will contain the nurse information and consent (File S3 in Supplementary) and the nurse assessment survey 1 (File S4 in Supplementary). The intervention group envelope will include, in addition to the above, the PCI (File S5 in Supplementary) and the nurse assessment survey 2 (File S6 in Supplementary).

The control group: an assessment will be completed following the current usual process. The nurse conducting the assessment will be asked to rate and justify the client’s complexity using judgment as usual and will not be prompted in any way.

The intervention group: an assessment, documentation and data collection will be carried out as for the control group with exception of the inclusion of survey 2. Once client assessment has been completed and away from client interaction, the nurse then completes the PCI and nurse survey 2 which comprises of questions pertaining to the PCI. The researcher will not lead or prompt the nurses.

### 2.9. Outcomes

The primary outcome of the RCT component is client complexity rating (low, moderate, or high) as measured using the PCI (intervention) or usual assessment nurse judgment alone (control).

The first secondary outcome is the addition of nursing care interventions after initial assessment. These are the required interventions for the client based on the assessment outcome and the detected level of client complexity. The nursing care interventions will be categorized as medication support, wound care, referrals and liaison and others. The second secondary outcome is the nursing care time added in minutes. This is the additional time that may be allocated to the delivery of the nursing care interventions for the clients following assessment and detected level of client complexity. 

Data will be collected using baseline and follow up surveys (File S1 and S2 in Supplementary) completed by participating nurses allocated to the intervention group pre- and post-PCI use. The survey will be administered electronically or via paper form. It will be supplemented with field notes and observations recorded by the researcher throughout the study. Any focus groups or interviews conducted will be recorded for transcription.

### 2.10. Data Analysis

Statistical analysis will be carried out in IBM SPSS Statistics Version 24 (IBM Corp, New York, NY, USA). For quantitative data analysis, statistical significance will be indicated by *p* < 0.05.

Participant characteristics and survey data will be analyzed and presented using summary descriptive statistics. Statistics will include mean and standard deviation for continuous data, median and interquartile range for ordinal data and percentage and 95% confidence interval for nominal data. Comparative univariate statistics will be conducted to test for differences between control and intervention characteristics. Parametric and or non-parametric statistical tests will be used to compare the groups.

Currently the PCI has no cut-offs for the level of complexity ratings. Preliminary data will be used in a receiver operating characteristics (ROC) analysis to determine complexity rating cut-offs (categorized as low, moderate, or high) with usual care complexity rating as the criterion. Categorial level of complexity rating as a primary outcome will be compared between control and intervention groups using the Chi-square independence test.

For the first secondary outcome, modification of nursing interventions (addition or subtraction of nursing interventions), between group comparisons will be performed on distribution of intervention categories. The second secondary outcome, clinical time will be compared between the study groups using ordinal regression. Clinical time as the dependent three level categorical variable. Age and nurse’ years of community experience will be covariates, while gender, reason for referral, group allocation (intervention or control) and level of complexity rating will be factors in the regression analysis.

Qualitative data collection will be from surveys, additional information regarding nursing interventions, nurse’s feedback, field notes and observations recorded by the researcher. Audio-recorded information from focus groups will be transcribed verbatim. Inductive thematic analysis has been chosen because this will ensure that themes emerge from the data (bottom-up approach). Analysis will be conducted using Braun and Clarke’s phases which include (1) data familiarization, (2) initial code generation, (3) theme search, (4) review of themes, (5) define and name themes [46].

### 2.11. Data Storage and Handling

Individual participant identifiers will be removed and replaced with a study participant ID prior to data entry into an electronic study database. The database will be kept separate from the assessments that form the client’s hospital medical record. The electronic files will be stored on a central secure drive at Bendigo Health and backed up on a central secure drive at Latrobe University established for research purposes. Hardcopy data will be stored in a locked filing cabinet at Bendigo Health. All data will be retained for seven years post completion as per the National Statement on Ethical Conduct in Human Research (NHMRC) ethics policy on clinical trials.

## 3. Expected Results

The primary objective of this trial is to enhance the detection of complexity of care for older people in the community. Aligned with this objective, using a validated complexity tool, such as the PCI, in addition to current assessment may complement nurses’ clinical judgment by providing an objective measure. Using a validated complexity tool may reduce assessment variations across staff undertaking this task, potentially increasing the reliability of complexity detection. Detection of complexity is currently dependent upon nurses’ clinical judgment, skills and experience to bring all the aspects of the assessments together and then determine care coordination, referrals and time that is required to deliver the care.

The secondary objective is to explore resource allocation. Embedding a validated tool into usual practice will not only standardize the approach to detecting complexity, it has the potential to streamline care coordination and documentation of care planning. The tool could prompt early referral discussions with the client and be used to summarize the strategies a client uses or wish to consider. This will in turn reduce duplication of referrals while ensuring care is delivered at the right time with the right resources that enable older clients to remain at home.

Nurses working in the community have a broad range of skills, level of experience and local knowledge that contributes to the detection of complexity in an objective way. The longer nurses work in the community, the more knowledge, experience, and skill is gained. Embedding the PCI into the service will help to bridge the gap in the nurse’s knowledge and experience providing an objective tool to support decisions making and clinical judgment.

## 4. Discussion

There are several clinical terms that identify an older person as requiring complex care or support, for example, multimorbidity and frailty [25,47]. These terms generally exclude the psychosocial care aspects of how a person lives which may be as important as physical or clinical care. Collaboration of multidisciplinary services can be impacted by the absence of person-centered care and a comprehensive approach to complex care [30]. This protocol describes a trial to enhance nurses’ clinical judgment, skills and experience working with older people.

The interface between healthcare and aged care continues to be a challenge in a large geographical country like Australia where there is a limited workforce to meet the care demands of an ageing population [44]. The Australian Royal Commission into Aged Care Quality and Safety which was concluded in 2021, highlighted issues of neglect and suboptimal care for older people [41]. The Royal Commission recommended that older people should receive timely care tailored to their individual needs and be provided with choice regard to their care. It also identified the need for an integrated system that supports wellbeing for older people, considering the importance of collaboration in enhancing continuity of care and ageing in place [30]. The report also highlights that whilst most aged care funds are spent on residential aged care, over two-thirds of our older people are cared for at home. Information regarding the services or supports available is difficult to find or navigate for older people and their families was also highlighted in the report. The comprehensive assessment conducted by nurses and their use of clinical judgment could be enhanced using an objective tool to determine the care needs required and assist the older person to understand what is available for them.

The United Kingdom is also working towards a collaborative approach through removing barriers to ensure that social care is considered, and collaboration is fostered. When considering the needs of the older person there needs to be a holistic approach that includes multimorbidity, frailty, social care needs. Understanding the whole person and working together to achieve outcomes that are value-based rather than medically based could provide a more coordinated health care system.

Using an instrument such as the PCI to support clinical judgment links all aspects of an older person which can then inform options. Clinical judgment and local knowledge of nurses is important to be able to assist with navigating a complex system and assist in reducing the barriers to receiving the care they require. The PCI was developed in Wales across four community nursing services. The demographic of the services and the clients in the service were determined to be like those in the Australian context. Using a concept mapping methodology, the factors impacting complexity were determined. Rasch analysis was used to establish the validity and reliability of each factor according to the individual psychometric properties. Each factor was determined to be valid and reliably impact the complexity of client needs [2]. Having a standardized tool such as the PCI that explores all the factors of a client’s situation could improve the time allocated to care provided and referrals completed. Care Coordination may be improved through providing earlier referrals thus engaging older people, their families, and other services earlier in their care journey.

The increasing complexity of clients in the community is a well-documented phenomenon and will continue to increase with Australian Government strategies to improve care to support people to remain at home for longer. The detection of complexity is important to enhancing nurses’ clinical judgment of complexity and facilitate the holistic delivery of appropriate care for older clients in a timely manner. Clinical judgment combined with the current assessment model could be enhanced using a tool that ensures all aspects of complexity are considered to determine additional supports a person may require.

## 5. Conclusions

This protocol outlines the study to enhance the detection of complexity by nurses delivering care for community-dwelling older people in the regional Australian context. The RCT will examine whether the PCI in addition to usual care may enhance the detection of the complexity of clients aged 65 and over. Focus groups will explore complexity from a multiple disciplinary perspective. The findings could benefit community nursing services across Australia and inform recommendations to improve the collaborative approach to care.

## Data Availability

Not applicable.

## Supplementary Materials

The following are available online at https://www.mdpi.com/article/10.3390/mps4040070/s1.
