# Supplementary material for: Improving Detection of Client Complexity in the Community (Impact): A Study Protocol of a Pragmatic Randomized Controlled Trial
Source: Methods Protoc. 2021 Oct 6;4(4):70. doi: 10.3390/mps4040070 (PMC8544575; doi:10.3390/mps4040070)
Supplement: Supplementary file 1 [file mps-04-00070-s001.zip › mps-1389208-supplementary.pdf]

File S1

A Pre pilot survey

Nurse code		
Total No. years in Nursing	RN:	EN:
No. years in the Community		
Age in years		

This survey will be completed at the start of the pilot in July and then again at the end in September. The intent is to gain additional information regarding the detection of complexity. Your response to these questions is appreciated.

On a scale of 1(not comfortable at all) to 5 (very comfortable)

Question	1	2	3	4	5
How comfortable do you feel in detecting patient complexity?					
How comfortable do you feel in adjusting interventions according to complexity?					
How comfortable do you feel in adjusting the time allocated according to complexity?					

You are currently using clinical judgement to detect complexity. What are the factors that you consider when thinking about patient complexity?
What are the barriers to detecting complexity?
What are the enablers to detecting complexity?

File S2

Post pilot survey

Nurse code	

You have now been involved in assessing patient complexity with and/or without the PCI. The intent is to gain additional information regarding the detection of complexity. Your response to these questions is appreciated. On a scale of 1(not comfortable at all) to 5 (very comfortable)

Question	1	2	3	4	5
How comfortable do you feel in detecting patient complexity?					
How comfortable do you feel in adjusting interventions according to complexity?					
How comfortable do you feel in adjusting the time allocated according to complexity?					
How comfortable do you feel using the PCI?					
How comfortable do you feel that using the PCI can support your decision regarding patient complexity?					

You are currently using clinical judgement to detect complexity; you may also consider complexity differently now. What are the factors that you consider when thinking about patient complexity?
What are the barriers to detecting complexity using the PCI?
What are the enablers to detecting complexity using the PCI?

File S3

Information and consent

BENDIGO HEALTH

Improving Quality of Care Through Detection of Complexity Amongst Older People in a Community Setting: Pilot Randomised Controlled Trial Protocol.

Participant Information Sheet

All nursing staff are invited to complete this short survey regarding complexity of patients and referrals completed.

Community patients are becoming increasingly complex. This is increasing the requirements of care and support to remain in their homes for longer. Currently complexity is detected using clinical judgment. There is no consistent method that is objective and links to time and resources allocated to patients.

The aim of this quality improvement activity is to explore the impact of the PCI to detect patient complexity and the subsequent resource and time allocation for community dwelling patients aged 65 and over. It is anticipated that using PCI will enhance the detection of patient complexity and appropriate resource and time allocation.

Aggregated data may be used in a publication or conference presentation. When the survey analysis is complete, a summary will be provided to all staff.

Completion of this survey will take no more than 15 minutes of your time. Your participation is completely voluntary and any identifying information will be removed by an independent person prior to data analysis.

If, in the unlikely event that it may be possible for you to be identified because you belong to a small subgroup your data will be grouped with others to make the subgroup larger to avoid individuals being identified.

This survey has been approved by Community Services Quality Review Meeting and the Bendigo Health's Human Research Ethics Committee.

Your consent to participate in this survey is implied if you choose to complete this survey. Should you decline your relationship with other staff and the organisation will not be affected.

If you would like to discuss this survey, please email Jennifer Boak at jboak@bendigohealth.org.au.

If you have any concerns of an ethical nature in relation to this survey please contact Sally McCarthy, Research Manager at Bendigo Health on 5454 6412 or samcarthy@bendigohealth.org.au.

File S4

Assessment survey 1

Date of assessment:

Nurse code		Patient code	

Patient age

Patient Gender

Male	Female	other

Reason for referral: ______________________________

What nursing interventions were allocated for this visit?

Nursing intervention	Time

Based on the information you have gathered about this patient please rate their level complexity

Low complexity	Moderate complexity	High complexity

Please explain how you came to this rating

Were any nursing interventions allocated after your assessment? If so, what were they?

Nursing intervention	Time

What referrals have been completed as a result of your admission, what is your rationale?

Referral to?	Rationale for referral

File S5

Date ____________________ Patient Name ______________________________ I.D. ________________ PCI Patient Complexity Instrument (PCI)

THE COMMUNITY-BASED PATIENT COMPLEXITY INSTRUMENT (V.1)						
Score each column	**Engagement** Identification and agreement of personalised care plan for and with this patient	**Clinical** Scale and scope of physical and psychological nursing needs	**Social Contact** Support systems for this patient	**Family and Carer** Factors relating to key carers (usually family)	**Resources** Any resources required to meet holistic needs and goals for this patient	**Safety** How safely the care will be delivered for this patient in the current environment
5	This patient lacks capacity<br>Or does not agree to engage in a shared care plan	This patient requires constant supervision<br>and immediate access to nurse intervention to avoid harm or sustain life	This patient is isolated & vulnerable to immediate risk or harm	The carer or family is isolated & vulnerable to immediate risk or harm<br>Or there is no carer	Imminent crisis or failure to progress care plan, care plan in dispute<br>Or resources not available	Patient safety is unachievable by remaining in this environment
4	This patient has fluctuating capacity<br>Or chooses not to engage in some aspects of a care plan	This patient has changeable needs<br>and requires nurse supervision within a comprehensive plan of care	This patient has very limited family support, social connection or activities	There is carer or family strain	Urgent referral (same day) to any multi-disciplinary/sector/ agency team or member, or specialist service	Patient safety is unpredictable by remaining in this environment
3	This patient has capacity<br>but there is some patient difficulty with engaging in a shared care plan	This patient has established on-going need<br>and requires scheduled nursing team interventions	This patient has support, social contact or activities available within informal family or community	Carer or family coping strategies are in place,<br>including patient's dependents	Referral for any aspect of a multi-disciplinary/sector/ agency team or member	Environmental or other factors present difficulties for patient, carer or nurse which might impact on patient safety
2	This patient has capacity<br>and agrees to engage with a suggested shared care plan	This patient has stable or predictable need<br>and requires some nursing team intervention and support	This patient has regular family or social contact and support for daily activities	The carer or family has regular family or social contact and support	On-going nursing assessment, intervention & care planning	Minor factors from within the home or the external environment present easily manageable risks to patient safety
1	There is full patient-led engagement with shared care plan	This patient is self-caring and requires minimal nursing team support	This patient is fully independent<br>Or well supported by family or community	The carer or family is fully independent and requires minimal support<br>Or there is no need for carer support	Task-oriented, delegated nursing team care and support	There is no identifiable environmental or other apparent risk to the patient

TOTAL SCORE ______________________________

This document was authored by Dr Sue Thomas (Sept 2017). Please contact sue.thomas@southwales.ac.uk for any assistance with the use of this instrument.
www.pci.wales

File S6:

Nurse assessment survey 2

(Intervention group)

Date of assessment:

Nurse code		Patient code	

After completing the PCI how would you rate this client's level of complexity?

Low complexity	Moderate complexity	High complexity

If this is different to your previous rating, if yes, please explain the change

Were any nursing interventions allocated after your assessment? If so, what were they?

Nursing intervention	Time

What referrals have been completed as a result of your admission or review, what is your rationale?

Referral to?	Rationale for referral
